# Optically switched magnetism in photovoltaic perovskite CH_3_NH_3_(Mn:Pb)I_3_

**DOI:** 10.1038/ncomms13406

**Published:** 2016-11-24

**Authors:** B. Náfrádi, P. Szirmai, M. Spina, H. Lee, O. V. Yazyev, A. Arakcheeva, D. Chernyshov, M. Gibert, L. Forró, E. Horváth

**Affiliations:** 1 Laboratory of Physics of Complex Matter, Ecole Polytechnique Fédérale de Lausanne (EPFL), CH-1015 Lausanne, Switzerland; 2 Institute of Physics, Ecole Polytechnique Fédérale de Lausanne (EPFL), CH-1015 Lausanne, Switzerland; 3 Swiss-Norwegian Beam Lines, European Synchrotron Radiation Facility, 71 Avenue des Martyrs, F-38043 Grenoble Cedex 9, France; 4 DQMP—University of Geneva, 24 Quai Ernest Ansermet, CH-1211 Geneva 4, Switzerland

## Abstract

The demand for ever-increasing density of information storage and speed of manipulation boosts an intense search for new magnetic materials and novel ways of controlling the magnetic bit. Here, we report the synthesis of a ferromagnetic photovoltaic CH_3_NH_3_(Mn:Pb)I_3_ material in which the photo-excited electrons rapidly melt the local magnetic order through the Ruderman–Kittel–Kasuya–Yosida interactions without heating up the spin system. Our finding offers an alternative, very simple and efficient way of optical spin control, and opens an avenue for applications in low-power, light controlling magnetic devices.

## Abstract (web summary)

Functional behaviour can emerge in materials in which magnetic order is determined by the interplay of localised and itinerant magnetic interactions. Here the authors tune such magnetic order in a photovoltaic perovskite by tuning the electronic carrier concentration under visible light illumination.

## Graphical abstract

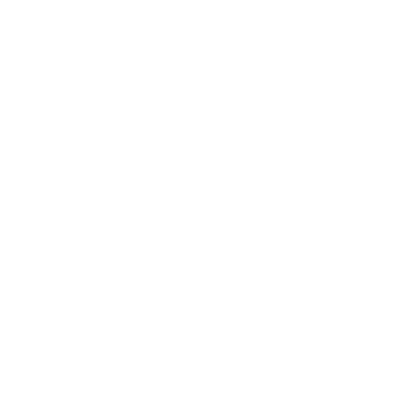

The mechanism of magnetic interactions and eventually the magnetic order in insulating and conducting materials are fundamentally different. Diluted localized magnetic (M) ions in insulating materials commonly interact over extended distances by the super-exchange (SE) interaction via atomic orbital bridges through nonmagnetic atoms, for example, oxygen, O. Common schemes for interactions in perovskite structures are the M–O–M or M–O–O–M-like bridges. The strength and sign (antiferromagnetic/ferromagnetic (AFM/FM)) of these interactions are determined by the geometry of the bonds. Thus, the *in situ* fine-tuning of the interactions is usually difficult because it would call for structural alterations. A limited continuous change is possible by application of pressure^1^. Discrete changes in the lattice are achieved by chemical modifications like replacing the bridging element with halides creating M–Cl–M, M–Br–M or M–I–M bonds^2^.

In conducting hosts the long-range double-exchange or the Ruderman–Kittel–Kasuya–Yosida (RKKY) interactions also come into play between the magnetic M ions. For the RKKY interaction, the key control parameters are the density of the localized moments and the density of itinerant electrons. The RKKY coupling strength oscillates between AFM or FM as a function of the M–M distance and of the size of the Fermi surface. These parameters, however, similarly to the case of the SE, are intrinsic to the system and *in situ* modifications are not feasible.

New magnetic materials and efficient, faster ways of controlling the magnetic bit are continuously searched for in order to sustain the needs for ever-increasing density and speed of information storage and manipulation^3^,^4^,^5^,^6^,^7^. Technologically relevant materials emerge when magnetic interactions of localized and itinerant spins are simultaneously present and compete in determining the ground state. This competition is usually controlled by the carrier concentration and a small external perturbation may result in an extremely large change, for instance, in resistivity. A well-known example is (La:Sr)MnO_3_ perovskite where at fine-tuned chemical substitutions ferromagnetic double-exchange interactions mediated by chemically doped electrons compete with the antiferromagnetic SE interaction of the parent insulating compound. Consequently, a metal-insulator transition and a ferromagnetic order develops^8^. Mechanical and electronic control of the carrier concentration and of the magnetic transition was also shown^1^,^9^,^10^.

Here we demonstrate an alternative way of controlling the competition of magnetic interactions between itinerant and localized electrons by using visible light illumination. By virtue of photodoping we modulate the carrier concentration and thus the magnetic order in the magnetic photovoltaic perovskite CH_3_NH_3_(Mn:Pb)I_3_. This method presents considerable advantages over chemical doping since it is continuously tuneable by light intensity, spatially addressable by moving the illuminating spot and, last but not least, provides a fast switching time (in the ns range required for relaxation of photo-excitations^11^,^12^). The observed optical melting of magnetism could be of practical importance, for example, in a magnetic thin film of a hard drive, where a small magnetic guide field will trigger a switching of the ferromagnetic moment into the opposite state via the light-induced magnetization melting. This kind of ferromagnetic moment reversal is rapid and represents several indisputable advantages over other optical means of manipulation of the magnetic state reported earlier^3^,^13^,^14^,^15^,^16^,^17^,^18^,^19^. The central ingredient is a high-efficiency photovoltaic material which orders magnetically. Taking advantage of the outstanding light-harvesting characteristics^20^ and chemical flexibility^21^ of the organometallic perovskite CH_3_NH_3_PbI_3_ (hereafter MAPbI_3_), which has recently triggered a breakthrough in the field of photovoltaics, we have developed a magnetic photovoltaic perovskite CH_3_NH_3_(Mn:Pb)I_3_ (hereafter MAMn:PbI_3_), (Fig. 1) by substituting 10% of Pb^2+^ ions with Mn^2+^ ions. This material provides a unique combination of ferromagnetism (*T*_C_=25 K) and high efficiency of photoelectron generation. It turns out that in our material these two properties are intimately coupled, thus optical control of magnetism is achieved. Furthermore, we expect this mechanism to be universally present in other magnetic photovoltaics, as well.

## Results

### Magnetic properties in dark

The substitution of Mn^2+^ ions into the MAPbI_3_ perovskite network is revealed by synchrotron powder X-ray diffraction and energy-dispersive X-ray measurements (Supplementary Fig. 1, Supplementary Table 1, and Supplementary Fig. 2). Mn^2+^ ions in the host lattice are isoelectronic with Pb^2+^. Hence, they do not dope the system as also confirmed by our first-principles electronic structure calculations discussed below. The substituted sample is semiconducting in dark with few MΩcm resistivity similarly to the parent compound. Moreover, the high level of Mn substitution does not diminish the photocurrent (*I*_ph_) generation. A strong *I*_ph_ response is observed below 830 nm wavelength (Supplementary Fig. 3). It is worth mentioning that the optical gap decreased relative to the pristine material, which facilitates photovoltaic applications. The photocurrent and thus the carrier density can be fine-tuned by the incident light intensity in broad frequency and intensity ranges. The Mn substitution, however, dramatically modifies the magnetic properties of the system as seen by electron spin resonance (ESR) measurements performed in an exceptionally broad 9–315 GHz frequency range (Figs 2 and 3, Supplementary Figs 4–6)^22^,^23^. The pristine material is nonmagnetic, only ppm level of paramagnetic impurities could be detected. As expected, Mn substitution gives an easily observable signal. At low concentration, ESR shows well-resolved hyperfine lines indicating the uniform dispersion of Mn^2+^ ions^24^ (Supplementary Fig. 5). The MAMn:PbI_3_ sample shows a strong ESR signal (Fig. 2 and Supplementary Fig. 5) and most importantly, a ferromagnetic order developing below *T*_C_=25 K on cooling in dark. The ferromagnetic order causes a rapid shift of the resonant field, *B*_0_, and the broadening of the linewidth, Δ*B*, below *T*_C_ (Fig. 2a and Supplementary Fig. 6) which are sensitive measures of the magnetic interactions and the internal magnetic fields^25^. The absence of additional ESR lines in the entire 9–315 GHz frequency range indicates that the magnetic order is homogeneous, the MAMn:PbI_3_ material is free of secondary phases corroborating with the structural refinement.

Static magnetization measurements by SQUID (inset to Fig. 2a and Supplementary Fig. 7) confirm the observations of ESR. The ground state is ferromagnetic as spontaneous magnetization *M*_spontaneous_ appears below *T*_C_ in the same temperature range where the ESR line shifts and broadens. Moreover, a small magnetic hysteresis appears at low temperatures indicated by the finite remanent magnetization (Supplementary Fig. 7). The deviation of *M*_spontaneous_ from the mean-field description (Bloch's Law) in the *T*_C_ to 2*T*_C_ temperature range is characteristic to diluted magnetic systems indicating the phase homogeneity of the system. Temperature and field dependence of *M* also revealed dominant ferromagnetic correlations at high temperatures, and the presence of magnetocrystalline anisotropy, *K*_1_=380 × 10^4^ J m^−3^, below *T*_C_ (Supplementary Fig. 7). The appearance of ferromagnetic order stabilized by short-range SE interactions in the insulating sample at 10% doping levels indicates that both Mn–I–Mn and Mn–I–I–Mn interactions are active to exceed percolation limits^26^. It should be emphasized that the homogeneous magnetic ordering itself in this insulating photovoltaic perovskite is already a remarkable observation. Such ordering was extensively searched for in homogeneously diluted magnetic semiconductors, and unambiguously observed only in few cases^24^,^27^,^28^,^29^.

This surprising FM order is supported by a rigorous density functional theory (DFT) calculations (see the ‘Methods' section for calculation details). The model of MAMn:PbI_3_ was constructed starting from the experimentally determined low-temperature orthorhombic (*Pnma*) crystal structure of undoped material^30^, which was then extended to the 2 × 1 × 2 supercell. Two Pb atoms in the supercell were replaced by Mn atoms to allow investigation of the exchange interactions between Mn dopants. Overall, one Pb atom of eight was substituted, which corresponds closely to the 10% doping concentration of experimentally investigated samples. Three different arrangements of Mn dopants were studied and are shown in Supplementary Fig. 8.

The energy differences between the FM and AFM configurations are of the order of 10–20 meV, while the interaction sign varies across the studied models. We found that for the in-plane model (model 2 in Supplementary Fig. 8), the FM configuration is the ground state, which is 10.9 meV lower in energy compared with the AFM configuration. The density of states plot calculated for the charge-neutral configuration of in-plane model shows that Mn^2+^ impurities substituting Pb^2+^ ions do not give rise to charge-carrier doping and do not induce any mid-gap states (Fig. 2b). The FM interaction is the consequence of the strongly distorted orthorhombic perovskite structure with Mn–I–Mn bond angle significantly reduced to ∼150° (Fig. 2c,d).

### Melting ferromagnetic order by photoelectrons

Our major finding is a striking change of the magnetism when the sample is exposed to light illumination at wavelengths lower than the bandgap, *λ*_edge_=830 nm (Supplementary Fig. 3). Typical ESR absorption spectra taken by light-off and light-on (0.8 μW cm^−2^) at *T*=5 K are shown in Fig. 3c. The light-on spectrum is considerably narrower and of weaker intensity than the spectrum in dark. The difference between light-on and light-off signals is shown in orange. For the given light intensity, 25% of the initial spin-susceptibility disappears (χ_ESR_) on light exposure. In a broad range of illumination intensities, after a threshold value, one can observe a monotonous decrease of the FM part of the signal (Fig. 3a). Presumably, below the threshold the photoelectrons fill up some trap sites. At larger intensities, they start to melt the FM state. (The same tendency is observed for Δ*B* versus illumination intensity, see Supplementary Fig. 9). The change is completely reversible. As χ_ESR_ is directly proportional to the ferromagnetic volume, the results demonstrate that in a large part of the sample the ferromagnetic order is melted by light illumination. This effect could be closely followed in temperature, as well. The difference between the light-on and light-off signals both in Δ*B* and in χ_ESR_ vary up to *T*_C_ (Fig. 3b). The narrowing of Δ*B* in the remaining magnetic signal, only observed below *T*_C_ (Fig. 3 and Supplementary Fig. 10), is a consequence of the surface melting of the magnetic order, as it is not accompanied by change of *B*_0_. The ferromagnetic Δ*B* is a strong function of sample shape and size. The light is absorbed in the first few microns of the crystals^11^ where the FM is molten so the created magnetic core-shell structure effectively changes the morphology of the sample, thus Δ*B.* The observations shown in Fig. 3b allow us to exclude heating effect by the light-emitting diode (LED) which means that this is an athermal magnetic change induced by photo-excited conduction electrons in the insulating magnetic phase.

### Model of the melting of the ferromagnetic order

On the qualitative basis, one can interpret the light-induced melting of the magnetic structure as the competition between the SE- and the light-induced RKKY-interactions^31^. SE orders the entire sample magnetically in dark. It is known that halide bridges can mediate the interaction between localized Mn^2+^ moments by SE in insulating perovskite crystals^32^. Illumination creates conduction electrons that alter the spin order established by SE as described by the RKKY Hamiltonian^33^. This mechanism is generic to all insulating magnets, where a high-efficiency photoelectron generation is present.

Electrical transport measurements in MAMn:PbI_3_ support this qualitative interpretation. The highly crystalline insulating sample with MΩcm range and thermally activated resistivity (not shown) transforms even into a metal-like state by the low-intensity red light illumination in a broad temperature range promoting the RKKY interaction. The quadratic magnetoresistance together with the resistivity indicates that even at low temperatures the photoinduced free carrier concentration exceeds *n*∼2 × 10^17^ cm^−3^. Furthermore, the weak, negative magnetoresistance in 0–2.5 T range (above this field a positive, orbital contribution is observed) shows the coupling of conduction electrons to the magnetic moments.

The idea to model the melting of the FM order by photoelectrons was to consider the competition between the SE- and the RKKY-interactions. This has been performed by DFT calculations (see the ‘Methods' section for calculation details). Technically, the effect of photo-excited charge carriers was addressed by considering separately electron- and hole-doped models since excitons cannot be described by DFT. Upon doping the in-plane configuration (Supplementary Fig. 8) with 2.6 × 10^20^ cm^−3^ concentration charge carriers, the ground state changes from FM to AFM with relative energies of 20.4 and 10.9 meV for one hole and for one electron per supercell, respectively. The corresponding total and projected density of states plots for the doped models in their AFM state are shown in Supplementary Fig. 11. We have to mention that the carrier concentration used in the modelling is much higher than the measured photoelectron concentration, but the purpose of our calculations is to demonstrate the suppression of the FM order. In fact, calculations with an order of magnitude lower carrier concentrations gave qualitatively identical results.

## Discussion

As an outlook, the observed optical melting of magnetism could be of practical importance, for example, in a magnetic thin film of a hard drive, where a small magnetic guide field will trigger a switching of the ferromagnetic moment into the opposite state via the light-induced magnetization melting. Its principle is illustrated in Fig. 4. This kind of ferromagnetic moment reversal is rapid and represents several indisputable advantages over other optical means of manipulation of the magnetic state reported earlier^3^,^13^,^14^,^15^,^16^,^17^,^18^,^19^. It does not require high-power or femtosecond laser instrumentation, which, besides the complexity of the techniques, raises the stability issue due to photochemistry and fatigue coming from the high intensity and the rapid local thermal cycling of the material^16^. Our method needs only a low-power visible light source, providing isothermal switching, and a small magnetic guide-field to overcompensate the stray field of neighbouring bits. Although this is a simple and elegant method for magnetic data storage, it has never been discussed in literature, because magnetic photovoltaic materials have not been developed.

We have shown the extension of photovoltaics into magnetism by preparing a ferromagnetic MAMn:PbI_3_. It has been demonstrated that the high-efficiency photocurrent generation by low-power visible light illumination results in a melting of the ferromagnetic state and a small local field can set the direction of the magnetic moment. It should be emphasized that this mechanism is radically different from switching the orientation of magnetic domains–here the photoelectrons tune the local interaction between magnetic moments and thus change the magnetic ground state. This study provides the basis for the development of a new generation of magneto-optical data storage devices where the advantages of magnetic storage (long-term stability, high data density, non-volatile operation and re-writability) can be combined by the fast operation of optical addressing. Such a technology should be developed with thin films with higher *T*_*C*_ (which is by far a non-trivial challenge) where the total melting of the magnetism in MAMn:PbI_3_ could be achieved on illumination. Last but not least, this study highlights that besides photovoltaics, lasing and LED operation there is one more extraordinary feature of the CH_3_NH_3_PbI_3_ perovskite material.

## Methods

### Sample preparation

**Figure 1:**
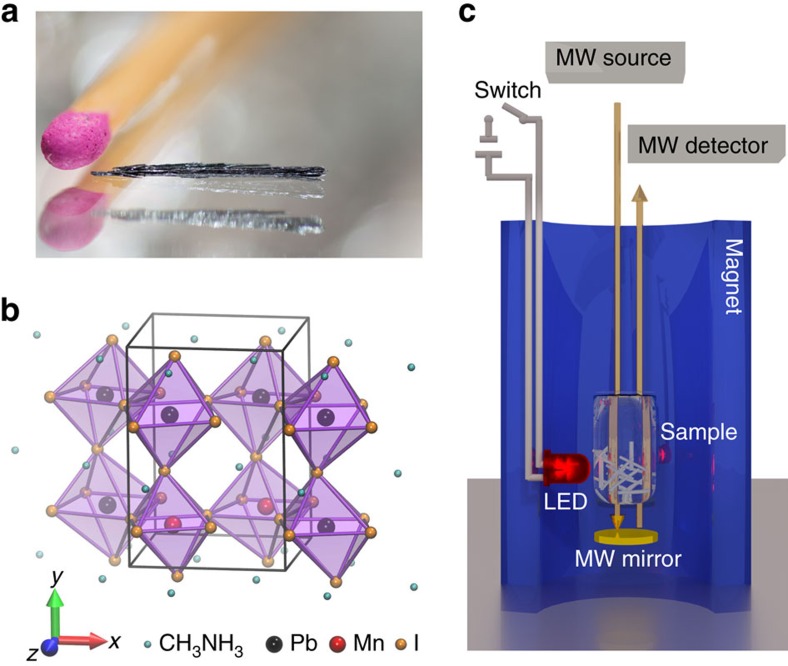
Sample and measurement configuration. (**a**) Photo of a typical CH_3_NH_3_(Mn:Pb)I_3_ crystal, 10–15 were assembled for the ESR measurement. (**b**) Sketch of the crystal structure of CH_3_NH_3_(Mn:Pb)I_3_. (**c**) The experimental configuration for the high-field ESR measurements showing the assembly of small crystals (sample). The absorption of the microwave field provided by the microwave source (MW source, up to 315 GHz) is monitored (MW detector) in resonant conditions in dark and under illumination in reflection geometry (MW mirror). The light source is a red (*λ*=655 nm, 4 μW cm^−2^) LED activated by an external switch (switch).

CH_3_NH_3_(Mn:Pb)I_3_ single crystals were prepared by precipitation from a concentrated aqueous solution of hydriodic acid (57 w% in H_2_O, 99.99% Sigma-Aldrich) containing lead (II) acetate trihydrate (99.999%, Acros Organics), manganese (II) acetate tetrahydrate (99.0%, Fluka) and a respective amount of CH_3_NH_2_ solution (40 w% in H_2_O, Sigma-Aldrich). The solubility of the Pb- and Mn-acetate provides indirect evidence of the homogeneous distribution of the Mn dopants. A constant 55–42 °C temperature gradient was applied to induce the saturation of the solute at the low temperature part of the solution^21^. Besides the formation of hundreds of submillimeter-sized crystallites (polycrystalline powder) large aggregates of long MAMn:PbI_3_ needle-like crystals with 5–20 mm length and 0.1 mm diameter were grown after 7 days (Fig. 1). Leaving the crystals in open air resulted in a silver-grey to green-yellow colour change. To prevent this unwanted reaction with moisture the as synthesized crystals were immediately transferred and kept in a desiccator prior to the measurements. Millimetre size undoped (CH_3_NH_3_PbI_3_) single crystals were also synthesized and kept as a reference material for qualitative analysis.

### Synchrotron X-ray powder diffraction (XRD)

Synchrotron XRD pattern of the CH_3_NH_3_(Mn:Pb)I_3_ sample was measured at room temperature at the Swiss—Norwegian beam lines of the European Synchrotron Radiation Facility (ESRF). The wavelength of the used synchrotron radiation was 0.9538 Å. All data were collected in the Debye–Scherrer geometry with a Dectris Pilatus2M detector. The sample-to-detector distance and the detector parameters were calibrated using a LaB_6_ NIST reference powder sample. CH_3_NH_3_(Mn:Pb)I_3_ powders were placed into 10 μm glass capillaries and mounted on a goniometric spinning head. For Rietveld refinement Jana crystallographic programme was used. Crystal structure was refined in *I*4/*mcm* tetragonal space group. Refined atomic parameters of Pb, I, C, and N are very similar to those published for CH_3_NH_3_PbI_3_ (ref. 34). In addition, H atoms were also localized. The XRD profile together with the results of the Rietveld profile fitting is shown in Supplementary Fig. 1 and in Supplementary Table 1. The synchrotron X-ray diffraction profiles revealed a sample without observable secondary phases or phase inhomogeneity.

### Scanning electron microscope

Scanning electron microscope images were taken with a MERLIN Zeiss electron microscope. Individual single needle-like crystallites were broken off from the rod-like bundles of MAMn:PbI_3_ for scanning electron microscope micrographs (Supplementary Fig. 2). Aluminium pucks were used for sample support. Conducting carbon tape served as electric contact between the sample and the support.

### Energy-dispersive X-ray spectroscopy (EDS)

The elemental composition of the MAMn:PbI_3_ crystallites were analysed by EDS (accelerating voltage of 8 kV, working distance of 8.5 mm). Samples were mounted on Al pucks with carbon tape with electrical contact to the surface also formed by carbon tape. The measurement was performed with an X-MAX EDS detector mounted at a 35° take-off angle with a SATW window. EDS spectra were obtained at a working distance of 8.5 mm with 8 keV accelerating voltage and a current held at 184 pA. 2,048 channels were used for the acquisitions, corresponding to energy of 5 eV per channel. Spectra were acquired over 1,573 s of live time with detector dead time averaging of 4% and a dwell time per pixel of 500 μs. Quantitative EDS analysis utilized Aztec software provided by Oxford Instrument Ltd.

To obtain information on the homogeneity of Mn substitution of the MAMn:PbI_3_ crystals, EDS were performed on several positions on the as-grown surface of the needle-like MAMn:PbI_3_ crystallites. For the purpose of gathering bulk information as well EDS spectrum were taken also on broken-off surfaces. These experiments systematically yield (Mn_0.1_Pb_0.9_)I_3_ stoichiometry indicating homogeneous Mn substitution.

### Electron spin resonance spectroscopy

Polycrystalline assembly of 10–15 rod-like MAMn:PbI_3_ samples with typical 1 × 0.1 × 0.1 mm are sealed in a quartz capillary. ESR at 9.4 GHz microwave frequency was performed on a Bruker X-band spectrometer. A conventional field modulation technique was employed with lock-in detection which results the first derivative of the ESR absorption spectra. Experiments in the mm-wave frequency range were performed on a home-built quasi-optical spectrometer operated at 75, 105, 157, 210 and 315 GHz frequencies in 0–16 T field range (Fig. 1). The spectral resolution of ESR is linearly proportional to the frequency, thus we extended the precision of ESR by about a factor 30 compared with X-band ESR technique. More details about the set-up can be found in refs 22, 23. A red LED was placed underneath the sample as a light source. Magnetic field strength at the sample position was calibrated against a KC_60_ standard sample. In contrast to the low-field ESR experiments, at millimetre-wave frequencies microwave power chopping was combined with lock-in detection. This detection scheme results directly the ESR absorption signal instead of its first derivative. The working principles of the two methods are shown in Supplementary Fig. 4.

Supplementary Fig. 5 compares pristine MAPbI_3_ with 1% and 10% substituted MAMn:PbI_3_ at room temperature. Pristine MAPbI_3_ crystals show no intrinsic ESR signal. Only low, ppm levels of paramagnetic impurity centres were observed (Fig. 3 and Supplementary Fig. 5). In contrast, Mn substitution to MAMn:PbI_3_ results in a strong ESR signal.

**Figure 2:**
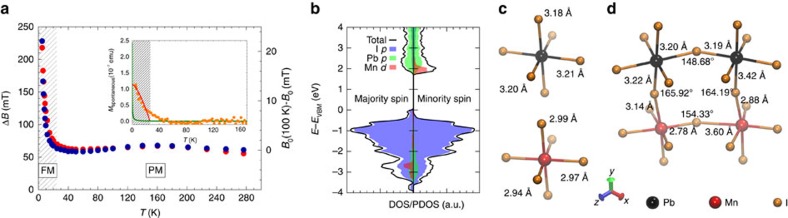
Magnetic properties of CH_3_NH_3_(Mn:Pb)I_3_ in dark. (**a**) ESR linewidth (red dots) and resonant field (blue dots, offset by a reference value *B*_0_) as a function of temperature recorded at 9.4 GHz. Their temperature-independent behaviour is characteristic for the paramagnetic phase (PM). The upturn below 25 K corresponds to the on-set of the FM phase. Inset: SQUID magnetometry of MAMn:PbI_3_. The temperature dependence of the spontaneous magnetization measured in 1.2 μT magnetic field shows a clear increase below *T*_C_. The red line represents the *M*_0_(1−(*T*/*T*_C_)^3/2^) temperature dependence given by Bloch's Law. (**b**) First-principles calculations of the atomic configurations and magnetic order show total density of states (DOS) and projected density of states (PDOS) calculated for the in-plane model of CH_3_NH_3_(Mn:Pb)I_3_ in its neutral FM configuration. (**c**) Calculated Pb–I and Mn–I distances for a single Mn dopant. (**d**) Calculated bond angles and bond distances for the I mediated super-exchange paths in the FM ground state of the in-plane model of CH_3_NH_3_(Mn:Pb)I_3_.

**Figure 3:**
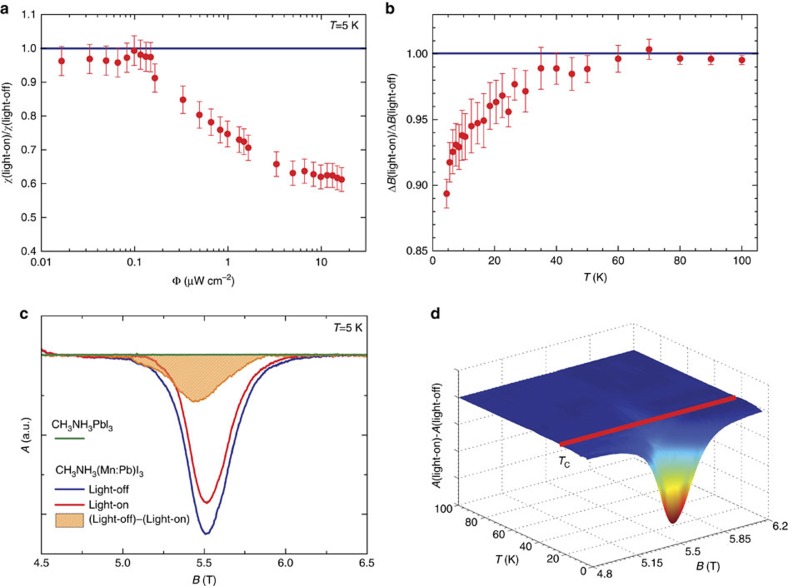
Illumination effect on the magnetic properties of CH_3_NH_3_(Mn:Pb)I_3_ measured by ESR. (**a**) The intensity change as the function of the illuminating red light intensity Φ at *T*=5 K. Above a threshold value, the FM part of the signal decreases monotonously. (**b**) Light-on ESR linewidth normalized to the linewidth in dark. The narrowing of the linewidth on illumination starts below *T*_C_. (**c**) ESR spectra at 157 GHz and 5 K of pristine CH_3_NH_3_PbI_3_ (green line–no signal), of CH_3_NH_3_(Mn:Pb)I_3_ in dark (blue line) coming from the FM phase and its reduction (red line) on visible light illumination. The difference between light-off and light-on ESR signals is shown in orange. The effect is accompanied by narrowing of the ESR linewidth on illumination. (**d**) Difference of the ESR intensities between the light-off and light-on measurements as a function of temperature. (The third axis shows the resonant field of the signal.) The intensity reduction on illumination is present only below *T*_C_=25 K, in the FM phase. Error bars represent the confidence interval of least square fits to the spectra.

**Figure 4:**
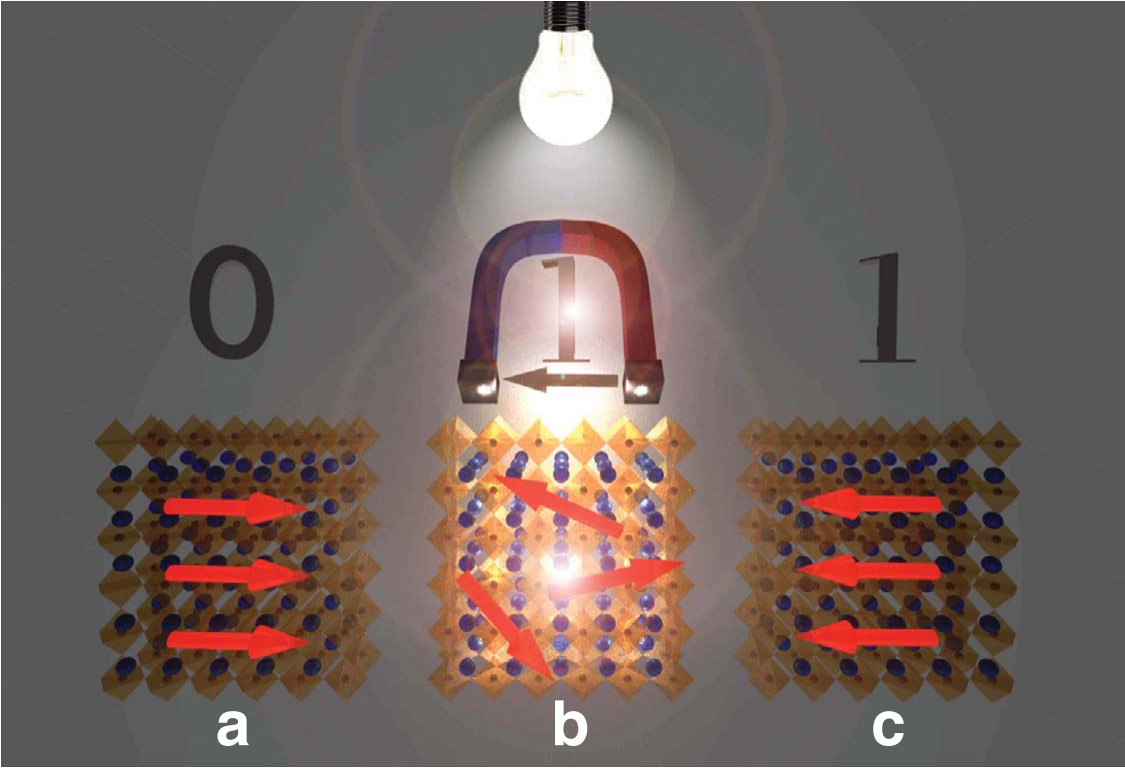
Schematic illustration of writing a magnetic bit. In the dark (**a**) the spin alignment corresponds to a given orientation of the magnetic moment in the FM state, representing a bit. On illumination (**b**) the FM order melts and a small magnetic field of the writing head will set the orientation of the magnetic moment once the light is switched off (**c**).

The presented ESR experiments prove that the magnetic transition is not driven by temperature change. ESR unambiguously demonstrates that we are not dealing with a temperature effect. By ESR at each temperature one obtains the spin susceptibility, ESR linewidth and resonance field simultaneously. All three parameters are strongly temperature-dependent as shown in Fig. 2 and Supplementary Fig. 6. Temperature change modifies all the three parameters concurrently. Our careful ESR experiments performed in dark provide us an internal thermometer. Increasing the temperature by ∼1 K would change all three aforementioned ESR parameters simultaneously. Switching on the light does not show this effect as demonstrated in Fig. 3. It changes the spin susceptibility, ESR linewidth and resonance field by an amount that corresponds to different temperature changes. This cannot be explained by a temperature effect. This shows unambiguously that we are not dealing with a temperature effect. Accordingly, the phenomenon we discovered is an athermal effect. Instead, we would like to point out that our ESR experiments are demonstrating the change of Curie temperature with photo-excitation. The spin susceptibility, measured by the ESR intensity, at *T*<*T*_C_ decreases by ∼25% on light illumination (Fig. 3b,d). This demonstrates the disappearance of 25% of the FM volume upon illumination. It means that in that 25% volume the *T*_C_ decreased from 25 K to below 5 K, the lowest temperature in our experiments.

The spectra at 1% Mn^2+^ concentration consist of two signals. One set of sextet lines and an about 50 mT broad line (Supplementary Fig. 5). The sextet signal is characteristic of a hyperfine splitting of ^55^Mn with *g*=2.001(1) *g*-factor and *A*_iso_=9.1 mT hyperfine coupling constant^24^. This spectrum corresponds to both allowed (sextet) and forbidden (broad component) hyperfine transitions between the Zeeman sublevels. It is characteristic to Mn^2+^ ions in octahedral crystal fields. Since strong forbidden transitions are observed, Mn^2+^ ions do not occupy strictly cubic sites, as strictly cubic centres have zero probability of forbidden transitions, rather distorted octahedral sites. The well-resolved hyperfine also testifies the homogeneous distribution of Mn ions in MAMn:PbI_3_ (ref. 24).

These ESR characteristics are in good agreement with both powder X-ray diffraction and DFT calculations showing distorted octahedral Mn coordination. The ESR spectra of MAMn:PbI_3_ at high Mn^2+^ concentration (10%) consist of one broad ESR line only. This is a common resonance of both allowed and forbidden transitions. We explain the uniformity of the *g*-factor by strong exchange narrowed spin–orbit interaction dominated linewidth of the Mn^2+^ ions. Following the calculations of ref. 35 and assuming a spin–orbit width contribution of the order of (Δ*g*/*g*)*J*, yields a value of the order of 100 K for exchange integral *J*.

The broad ESR and isotropic *g*-factor is strongly intrinsic for the system. We find no evidence of frequency dependence at high temperatures in the 9–315 GHz frequency range. The field- and temperature-independent Δ*B* and *B*_0_ is characteristic to exchange coupled paramagnetic insulators. Below 25 K, both Δ*B* and *B*_0_ acquires strong temperature dependence indicative of ferromagnetic ordering. The shift in *B*_0_ measures the temperature dependence of the internal ferromagnetic field of MAMn:PbI_3_. Δ*B* scales to *B*_0_ at all measured fields and temperatures (Fig. 2 and Supplementary Fig. 6) indicating an inhomogeneous broadening induced by spatial distribution of the local internal ferromagnetic field. The inhomogeneity of the local internal ferromagnetic field is partially of geometrical origin. The demagnetizing field of our irregularly shaped particles is inhomogeneous. Additionally, the statistical fluctuations of the Mn concentration across the sample also increase the inhomogeneity by modulating the strength of the ferromagnetic order.

The magnetic phase purity can be further confirmed by comparing the two described ESR method. The microwave chopping method (Supplementary Fig. 4b), which yields the integrated ESR signal, would reveal possible broad ESR signals. However, Fig. 3c,d show the absence of any broad magnetic impurity signals. The magnetic field modulation method (Supplementary Fig. 4a) would help to identify narrow signals with a linewidth in the order of the modulation. Supplementary Fig. 5f proves the absence of the narrow impurity signals as well.

We note here that the signal of itinerant electrons generated by the illumination is not detected, either. Detection of a so-called conduction ESR (CESR) line would be a major challenge (see, for example, refs 24, 36). The two main difficulties in the order of the importance are: (i) low Pauli spin susceptibility of a CESR signal, and (ii) the spin–orbit coupling provokes a broadening in the signal^37^,^38^.

In our system, the presence of conduction electrons can be excluded in dark, as MAMn:PbI_3_ is an insulator without light. We would only observe the CESR signal in the presence of photoexcited carriers on illumination. As seen in Fig. 3c and in Supplementary Fig. 5f, however, we do not observe the CESR on illumination. In our case, both issues of CESR detection are significant. The weak illumination results in the small spin-susceptibility of the generated conduction electrons. In fact, the expected spin susceptibility of the CESR (Pauli susceptibility) of the photoexcited state is 5–6 orders of magnitude smaller than the paramagnetic Mn^2+^ ESR signal. Furthermore, the large spin–orbit coupling broadens this small signal. These two effects prevent the observation of the CESR.

Furthermore, the precursor Mn-acetate used for the Mn substitution has markedly different ESR spectra from the substituted material, thus inclusions of Mn-acetate islands can be excluded, as well.

### SQUID magnetometry

SQUID magnetometry experiments reveal that the temperature dependence of spontaneous magnetization, the defining macroscopic property of ferromagnetism, appears below 50 K and dramatically enhances below 25 K (Fig. 2). The theoretical behaviour of the paramagnetic magnetization in the same conditions is shown by the green line. Clearly, the spontaneous magnetization is orders of magnitude greater compared with a paramagnetic magnetization, testifying the ferromagnetic order.

The mean-field theory of spontaneous magnetization is described by Bloch's Law, which states that *M*_spontaneous_∼1−(*T*/*T*_C_)^*α*^ with *α*=3/2 (red line in Fig. 2). However, deviations from the mean-field exponent are recurrent, for example, Iron and Nickel show critical exponents *α* of 0.34 and 0.51, respectively. Similarly, the spontaneous magnetization in MAMn:PbI_3_ deviate from the mean-field value (Fig. 2). The deviation from Bloch's Law is indicative of the presence of strong magnetocrystalline anisotropy. The primary source of magnetocrystalline anisotropy is the spin–orbit interaction, which is strong due to the involvement of Pb and I atoms.

In Supplementary Fig. 7a, we show the temperature dependence of the magnetization cooled in 1 T external field. In agreement with the appearance of the remanent magnetization in the zero field-cooled experiments, we find a Curie–Weiss temperature of *T*_CW_=14 K characteristic to predominant ferromagnetic correlations. At low temperatures, however, the magnetization is suppressed relative to the isotropic Curie–Weiss behaviour. This is characteristic to the presence of magnetocrystalline anisotropies in perfect agreement with the observed deviation of the spontaneous magnetization from the mean-field description.

The magnetic field dependence of the magnetization measured at *T*=2 K up to 7 T magnetic field (see Supplementary Fig. 7b) shows a steady increase of magnetization with about *H*_S_=2*K*_1_/*M*_S_=9 T saturating magnetic field. This again underlines the presence of magnetic anisotropy of *K*_1_=380 × 10^4^ J m^−3^ at *T*=2 K. Note that this value is in the same range as those found at room temperature in haematite (*K*_1_=120 × 10^4^ J m^−3^) and for YCo_5_ (*K*_1_=550 × 10^4^ J m^−3^).

Finally, the temperature dependence of the remanent magnetization measured by decreasing the magnetic field from 7 T shows small value in agreement with the magnetization isotherms. The temperature behaviour is similar to the behaviour of the spontaneous magnetization, and it increases below 50 K (inset of Fig. 2).

These SQUID experiments undoubtedly reveal the existence of magnetic order of our MAMn:PbI_3_ compound. It also shows high magnetic phase purity. No sign of additional magnetic or nonmagnetic phase was detected in perfect agreement with the multi-frequency ESR investigations.

### Photocurrent spectroscopy

For photocurrent spectra a low-intensity monochromatic light was selected by a MicroHR grid monochromator from a halogen lamp. The wavelength resolution (full width at half maximum; FWFM) of the 600 g mm^−1^ grating was 10 nm. The photo-excited current was measured by a two-terminal method at fixed bias voltage of 1 V while the wavelength was stepwise changed (Supplementary Fig. 3). Measurements were performed on pristine MAPbI_3_ and on MAMn:PbI_3_. The bandgap energy was determined by fitting a Fermi–Dirac distribution to the data. The resulting gap energies at room temperature are 783±1 nm and 829±1.4 nm for the MAPbI_3_ and MAMn:PbI_3_, respectively. The intrinsic width of the Fermi–Dirac distribution for both systems is thermally broadened. This indicates that the Mn substitution is homogeneous. Mn clustering would cause broadening of the band edge. It is also worth mentioning the strong, ∼46 nm upshift of the band edge on Mn substitution since the gap of MAMn:PbI_3_ is reduced relative to MAPbI_3,_ Mn substitution presents an alternative route to extend the light absorption range, hence increase photocell efficiencies. The temperature dependence of the photocarrier generation in 50–300 K temperature range was also studied in a closed-cycle cryostat equipped with an optical window (Supplementary Fig. 3). The gap energy increases by decreasing temperature due to thermal expansion, however, the photocarrier generation of MAMn:PbI_3_ remains effective down to the lowest studied temperatures.

### First-principles electronic structure calculations

To corroborate the experimental findings, we carried out first-principles electronic structure calculations in the framework of DFT^39^,^40^ as implemented in the Quantum ESPRESSO package^41^. The exchange-correlation energy is given by the Perdew–Burke–Ernzerhof generalized gradient approximation^42^ while the electron–ion interactions are treated by using the ultrasoft pseudopotentials^43^ which have been previously published^44^. Wave functions and charge densities are expanded using the plane-wave basis sets with kinetic energy cutoffs of 40 and 320 Ry, respectively. The Brillouin zone is sampled using 3 × 4 × 3 Monkhorst-Pack meshes of special **k**-points^45^. The plane-wave cutoffs and **k**-point meshes are chosen to ensure the convergence of total energies within 10 meV. When performing calculations on charged models, a compensating jellium background was introduced in order to avoid the spurious divergence of electrostatic energy^46^.

The models of Mn-doped CH_3_NH_3_PbI_3_ were constructed starting from the experimentally determined crystal structure of undoped material (orthorhombic phase, space group *Pnma*)^30^, which was then extended to the 2 × 1 × 2 supercell by doubling the lattice constants along the *a* and *c* directions. Two Pb atoms in the supercell were replaced by Mn atoms to allow investigating the exchange interactions between Mn dopants. Overall, one Pb atom of eight was substituted, which corresponds closely to the doping concentration of experimentally investigated samples (10%). Three different arrangements of Mn dopants, referred to as top, in-plane, and diagonal, are shown in Supplementary Fig. 8. Atomic coordinates of all these three configurations were optimized to the residual ionic forces smaller than 0.02 eV Å^−1^, whereas the lattice parameters were kept fixed. For each configuration both the FM and AFM arrangements of local magnetic moments of Mn atoms were investigated. Our calculations show that optimization of the internal atomic coordinates is crucial for reproducing the relative energies of FM and AFM configurations. Indeed, substitution of Mn atoms for Pb atoms leads to a pronounced lattice distortion around the Mn dopants due to different ionic sizes of Mn^2+^ and Pb^2+^. Specifically, the Mn–I distances are about 2.9 Å, whereas the Pb–I distances are about 3.2 Å (Fig. 2c,d).

For all considered arrangements of Mn dopants, the energy differences between the FM and AFM configurations are of the order of 10–20 meV. We found that for model 2 (in-plane, Supplementary Fig. 8), the FM configuration is the ground state, which is 10.9 meV lower in energy compared with the AFM configuration. Due to intrinsic limitations of DFT calculations, the effect of photoexcited charge carriers was addressed by considering separately electron- and hole-doped models. One has to emphasize that the DFT calculations correspond to a 0 K case and fixed number of photoelectrons. At finite temperatures and variable carrier density between the FM and AFM configurations it is reasonable to expect a paramagnetic state as seen in the experiment.

### DC resistivity and magnetotransport under illumination

DC resistivity and magnetotransport under illumination were performed with the same light conditions as the ESR experiments. Resistivity and magnetoresistance were measured in a standard 4-terminal configuration in the 5–300 K temperature and 0–16 T magnetic field range. In dark, the resistivity of the samples is in the MΩcm range and show thermally activated character (not shown). Under red light illumination, the resistivity monotonically drops by lowering temperature. At the structural transition temperature around 150 K, however, the resistivity discontinuously jumps. Magnetoresistance at low temperatures increases quadratically by increasing magnetic field. In the carrier/exciton ratio study of D'Innocenzo *et al*.^47^, it was suggested that free charge carriers are predominant in perovskite solar cells at room temperature. Hence, the perovskites possess large built-in fields which can effectively drift photogenerated carriers to avoid charge recombination. These are in perfect agreement with our magnetoresistance and photocurrent spectroscopy measurements. The resistivity decreases by a factor more than 400 by cooling from 300 to 30 K. This clearly indicates the presence of free carriers at temperatures relevant to FM melting. The photocurrent generation is an active process. Also, the photocurrent spectra at 50 K clearly shows that free carriers are readily excited in our experiments.

Our experiments were performed under continuous illumination, which implies a constant number (time-independent after a few fs transient) of out-of-equilibrium photoexcited carriers next to the thermalized free carriers and excitons.

### Data availability

The data that support the findings of this study are available from the corresponding authors upon request.

## Additional information

**How to cite this article:** Náfrádi, B. *et al*. Optically switched magnetism in photovoltaic perovskite CH_3_NH_3_(Mn:Pb)I_3_. *Nat. Commun.* **7,** 13406 doi: 10.1038/ncomms13406 (2016).

**Publisher's note**: Springer Nature remains neutral with regard to jurisdictional claims in published maps and institutional affiliations.

## Supplementary Material

Supplementary Information Supplementary Figures 1-11 and Supplementary Table 1.
