# Supplementary material for: Optically switched magnetism in photovoltaic perovskite CH3NH3(Mn:Pb)I3
Source: Nat Commun. 2016 Nov 24;7:13406. doi: 10.1038/ncomms13406 (PMC5123013; doi:10.1038/ncomms13406)
Supplement: Supplementary Information — Supplementary Figures 1-11 and Supplementary Table 1. [file ncomms13406-s1.pdf]

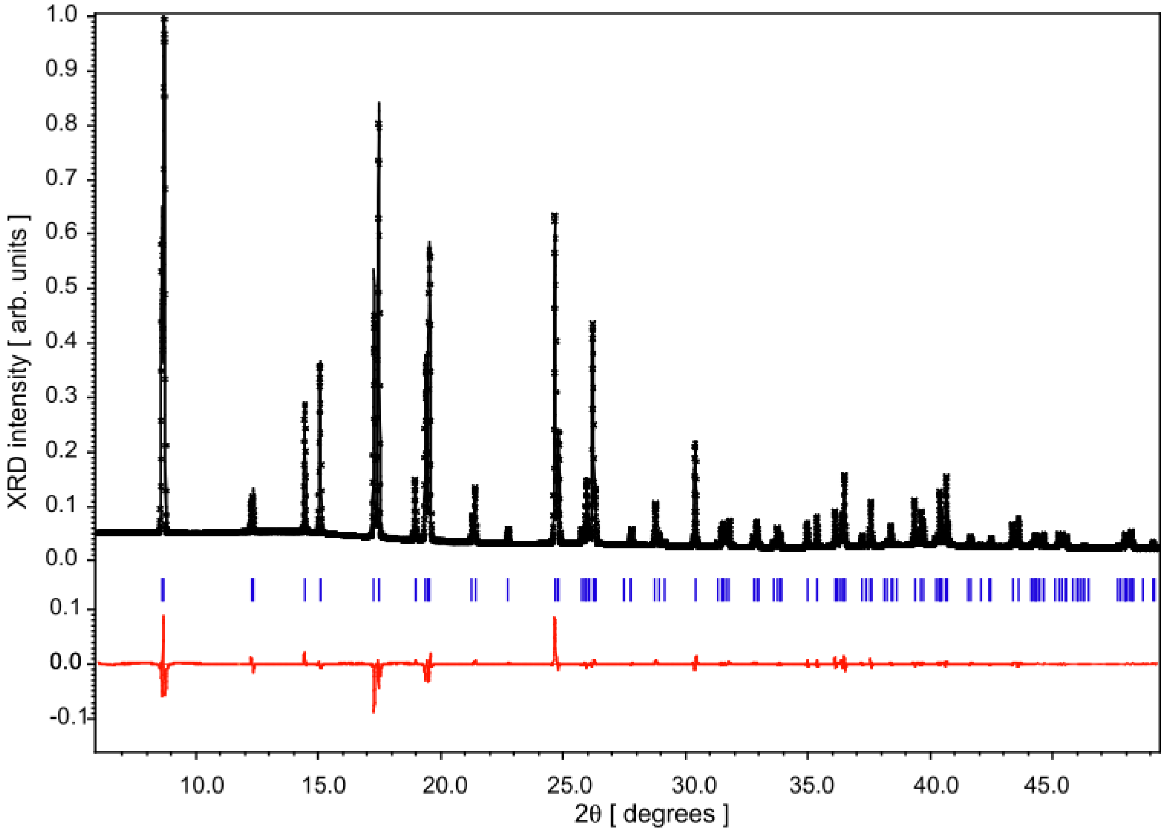

Supplementary Figure 1 - Synchrotron powder X-ray diffraction. Room temperature synchrotron powder X-ray profile of MAMn:PbI_3 (wavelength of the synchrotron radiation is equal to 0.9538 Å). Stars and solid and thin lines (black) correspond to experimental data and calculation, respectively. Deviation from the fit is shown in red. Strips (blue) indicate positions of the Bragg reflections. The Rietveld refinement shows a perfectly single phased material: MAMn:PbI_3 sample is free of PbI_2, Mn clusters or any other impurity.

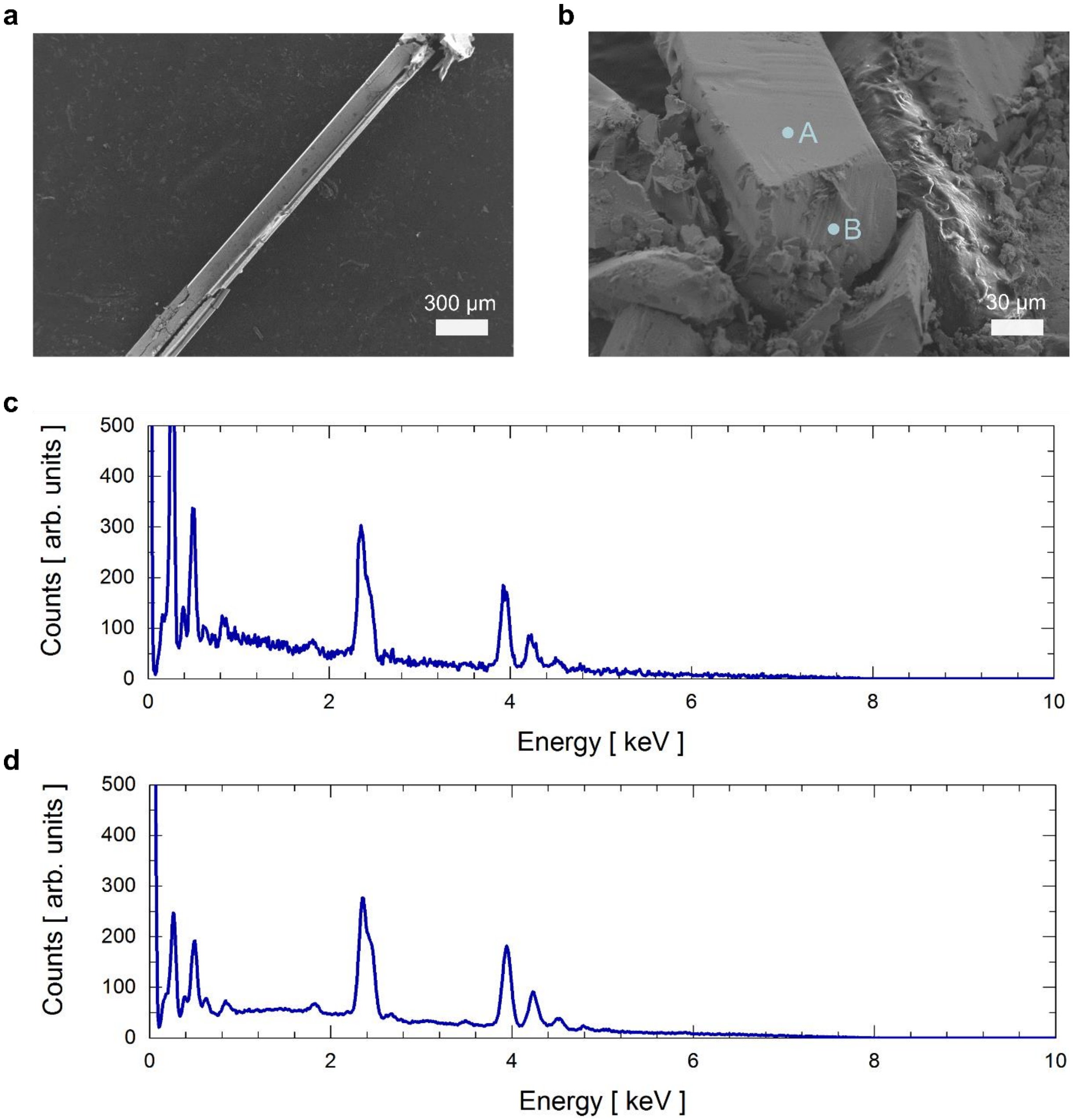

Supplementary Figure 2 - Energy dispersive X-ray spectroscopy. (a) SEM micrograph of a typical MAMn:PbI_3 single crystal of several mm in length and 100×100 μm^2 in cross-section. **(b)** Zoom on a broken section of the needle shown in **a**. A and B are the positions where the EDS spectra were obtained. **(c-d)** EDS sum spectra obtained at the as-grown and broken surfaces indicated by A (**c**) and B (**d**), respectively in **b**. The stoichiometry at both regions is $Pb_{0.9}Mn_{0.1}I_3$, testifying the homogeneous bulk substitution of Mn ions.

2

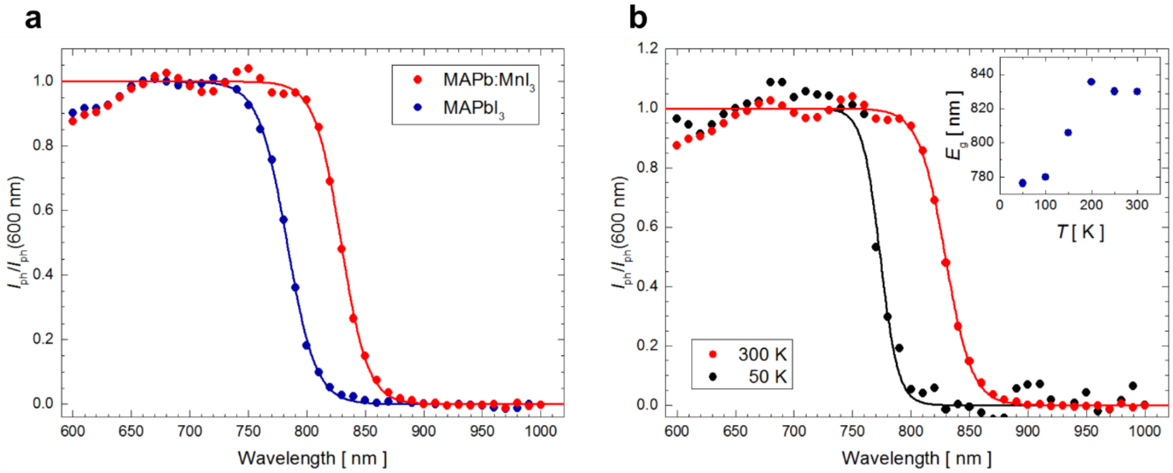

Supplementary Figure 3 - Photocurrent spectra. (a) Photocurrent of MAMn:PbI_3 (red symbols) and $MAPbI_3$ (blue symbols) at fixed bias voltage of 1 V measured as a function of photon energy at 300 K. The strong photocurrent generation above the optical band gap of ~830 nm of MAMn:PbI_3 is red shifted by about 46 nm relative to that of the pristine $MAPbI_3$ material (783 nm). Lines are fits to modelling the band edge by the Fermi-Dirac distribution and its thermal broadening. **(b)** Comparison of the T=50 K (black) and T=300 K (red) photocurrent spectra of MAMn:PbI_3. Inset shows the temperature evolution of the bandgap (E_g) obtained from photocurrent spectroscopy.

3

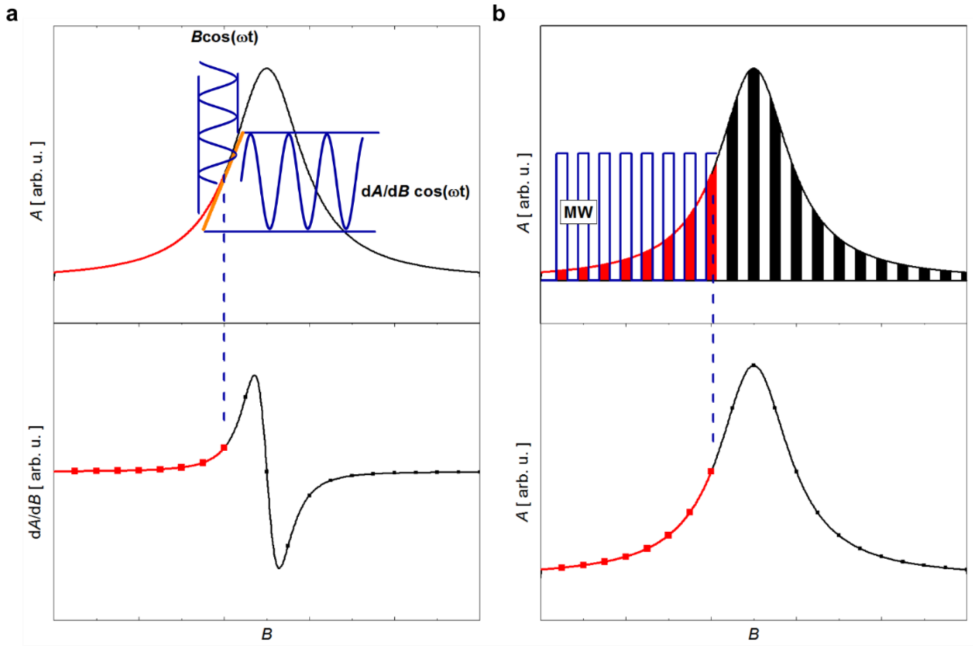

Supplementary Figure 4 - Basic principle of ESR signal detection. (a) Conventional magnetic field modulation used in 9.4 GHz ESR experiments. Upper curve represents the ESR absorption A as a function of magnetic field B. The modulation magnetic field $B\times\cos(\omega t)$ and the resulting modulated microwave absorption power $\mathrm{d}A/\mathrm{d}B\times\cos(\omega t)$ are also illustrated. Lower panel depicts the *first derivative* $\mathrm{d}A/\mathrm{d}B$ signal of the ESR absorption line A after lock-in detection. **(b)** Microwave (MW) chopping detection used for 105 and 157 GHz ESR experiments. The microwave radiation is periodically switched on/off (blue line). Accordingly, the ESR absorption signal is modulated as shown by the red shaded area. The lower panel presents the resulting absorption ESR line A after lock-in detection.

4

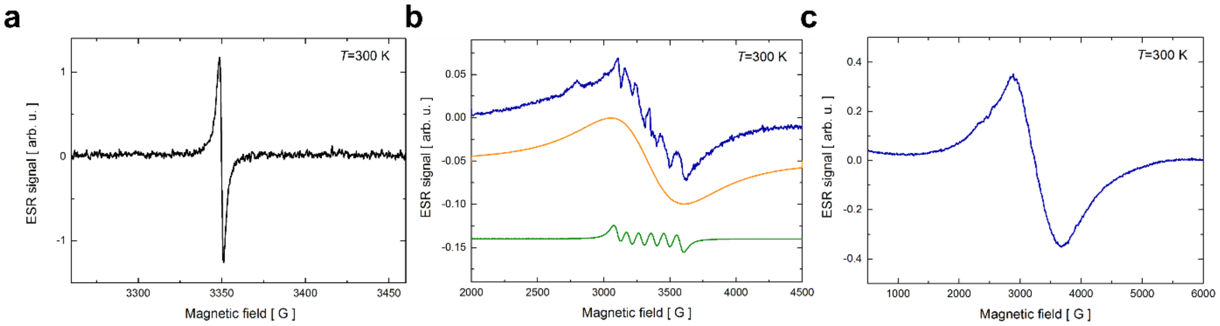

Supplementary Figure 5 – Room-temperature 9.4 GHz ESR spectra. (a) Spectrum of pristine $MAPbI_3$. Only a weak paramagnetic impurity signal is observed characteristic of ppm level defect concentration. **(b)** Spectra of $MAMn:PbI_3$ with low (~1%) Mn concentration. A forbidden hyperfine signal (orange) and allowed hyperfine sextet line (green) of the Mn^{2+} reproduce the observed signal well (blue). The well-resolved hyperfine structure indicates the homogeneous dispersion of the Mn^{2+} ions. **(c)** Spectrum of $MAMn:PbI_3$ with high (10%) Mn concentration.

5

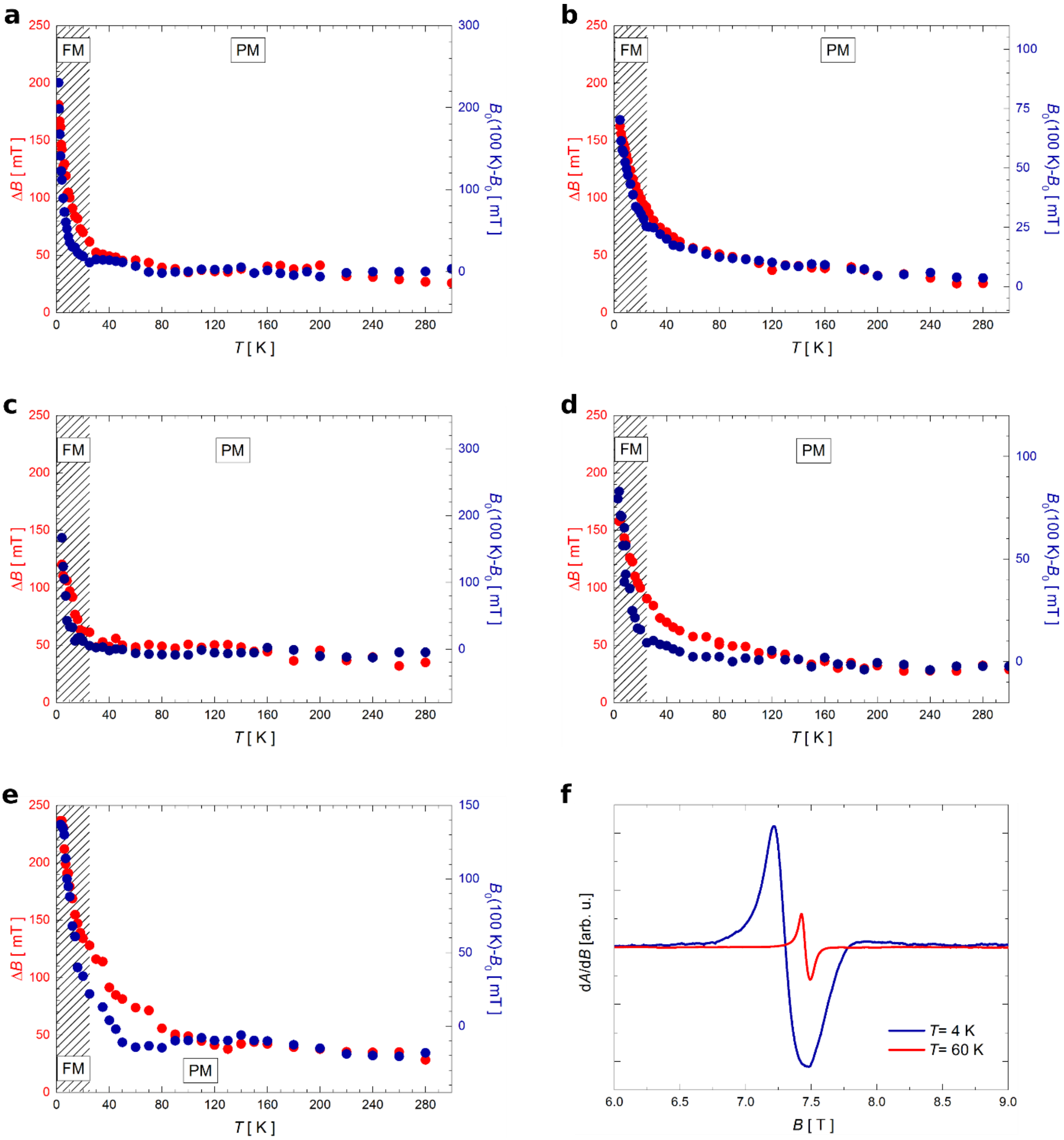

Supplementary Figure 6 - Multifrequency ESR properties of MAMn:PbI$_3$. (a-e) ESR at 75 (**a**), 105 (**b**), 157 (**c**), 210 (**d**), and 315 GHz (**e**) frequencies were measured as a function of temperature. The temperature dependence of the linewidth (red) scales with the temperature dependence of the ESR shift B_0(100 K)-B_0 (blue) showing that both quantities measure the local dipole field distribution of the polycrystalline ferromagnetic material. FM and PM show the ferromagnetic (shaded area) and paramagnetic state, respectively. (**f**) Comparison of the first-derivative ESR spectra measured below (T=4 K) and above T_C (T=60 K). Absence of narrow ESR components below T_C proves the high magnetic phase purity.

6

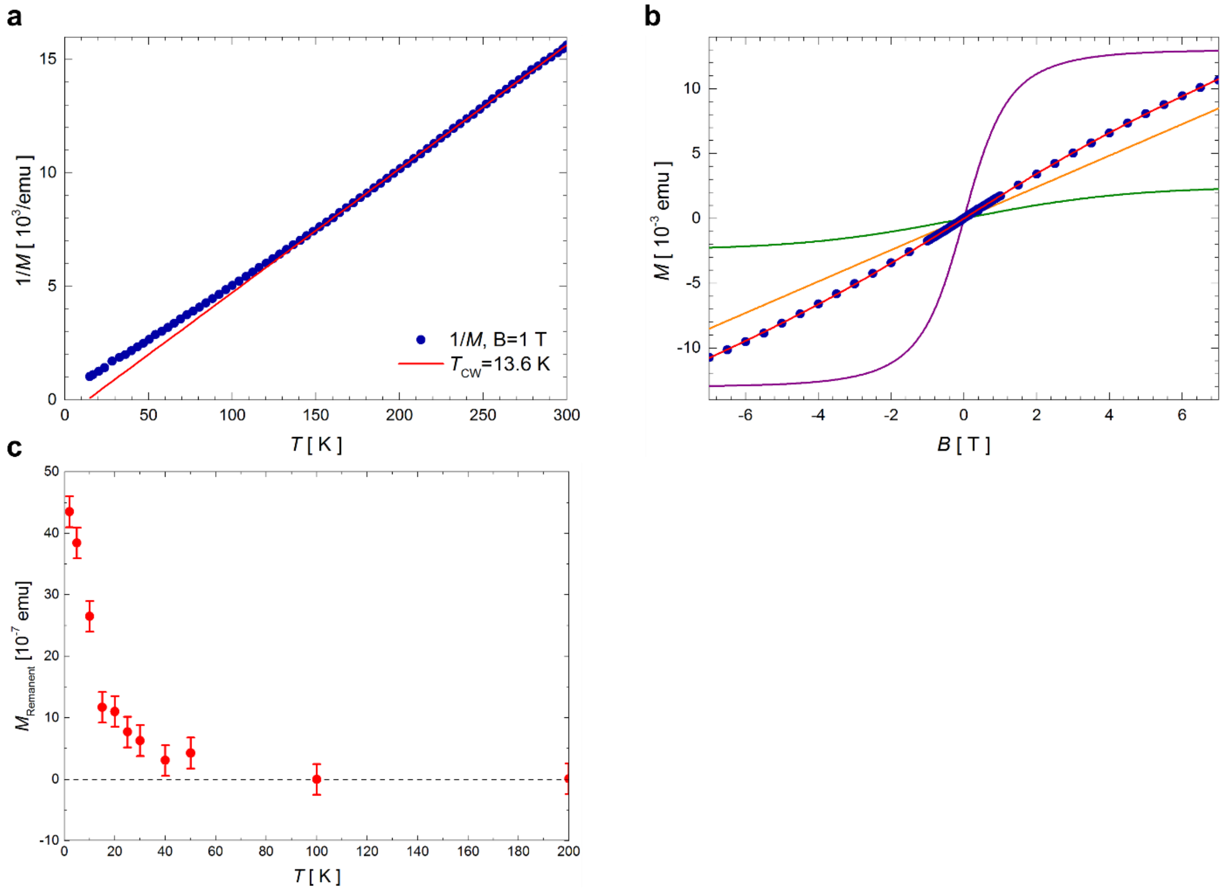

Supplementary Figure 7- SQUID magnetometry of MAMn:PbI_3. (a) Temperature dependence of 1/*M* cooled in 1 T magnetic field. Line presents the Curie-Weiss fit which reveals predominant ferromagnetic correlations with a Curie-Weiss temperature of T_{CW}=13.6 K. **(b)** Magnetization measured a *T*=2 K after a field cooled process in 7 T. The field dependence is remarkably well described by (red line) a ferromagnetic powder with K_1=380×10^4 J/m^3 (orange) and with a small magnetic domain contribution (green). The observed behaviour is clearly distinct from a paramagnet case (purple). **(c)** Remanent magnetization as a function of temperature. Error bars represent the confidence interval of least square fits to the *M*(*H*) curves.

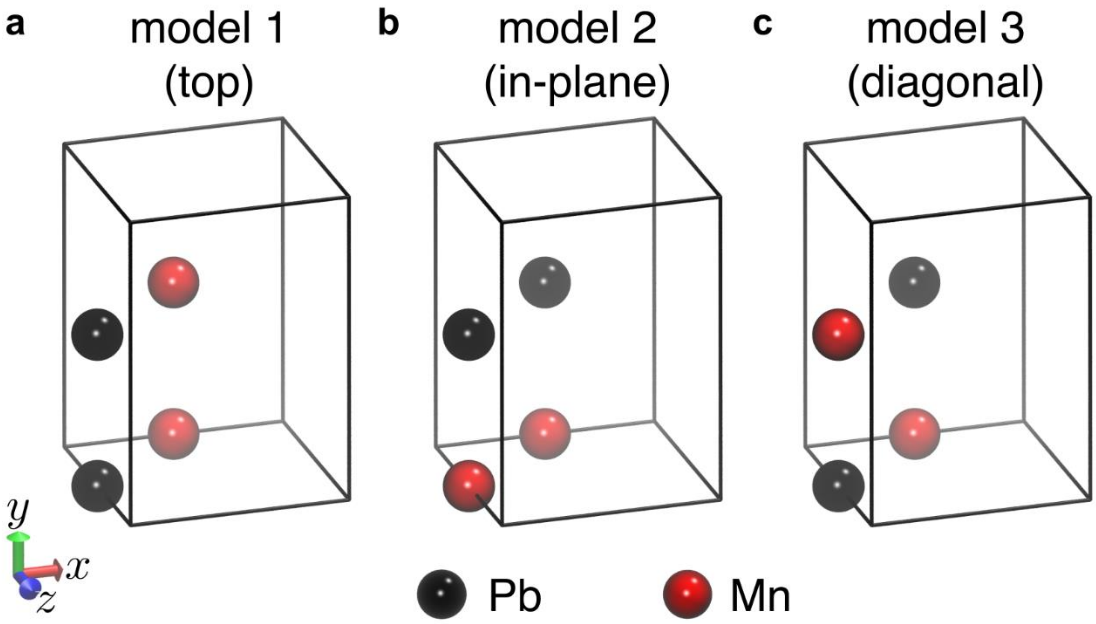

Supplementary Figure 8 – Models of the Pb and Mn distributions in MAMn:PbI$_3$ Schematic drawings of three models of MAMn:PbI$_3$ containing pairs of Mn dopants in close proximity to each other in the 2×1×2 supercell studied by means of first-principles calculations. The three configurations investigated are referred to as top (**a**), in-plane (**b**), and diagonal (**c**). For clarity, only Pb (black) or Mn (red) atoms are shown and the unit cell of the undoped orthorhombic-phase MAPbI$_3$ is indicated by black lines.

18

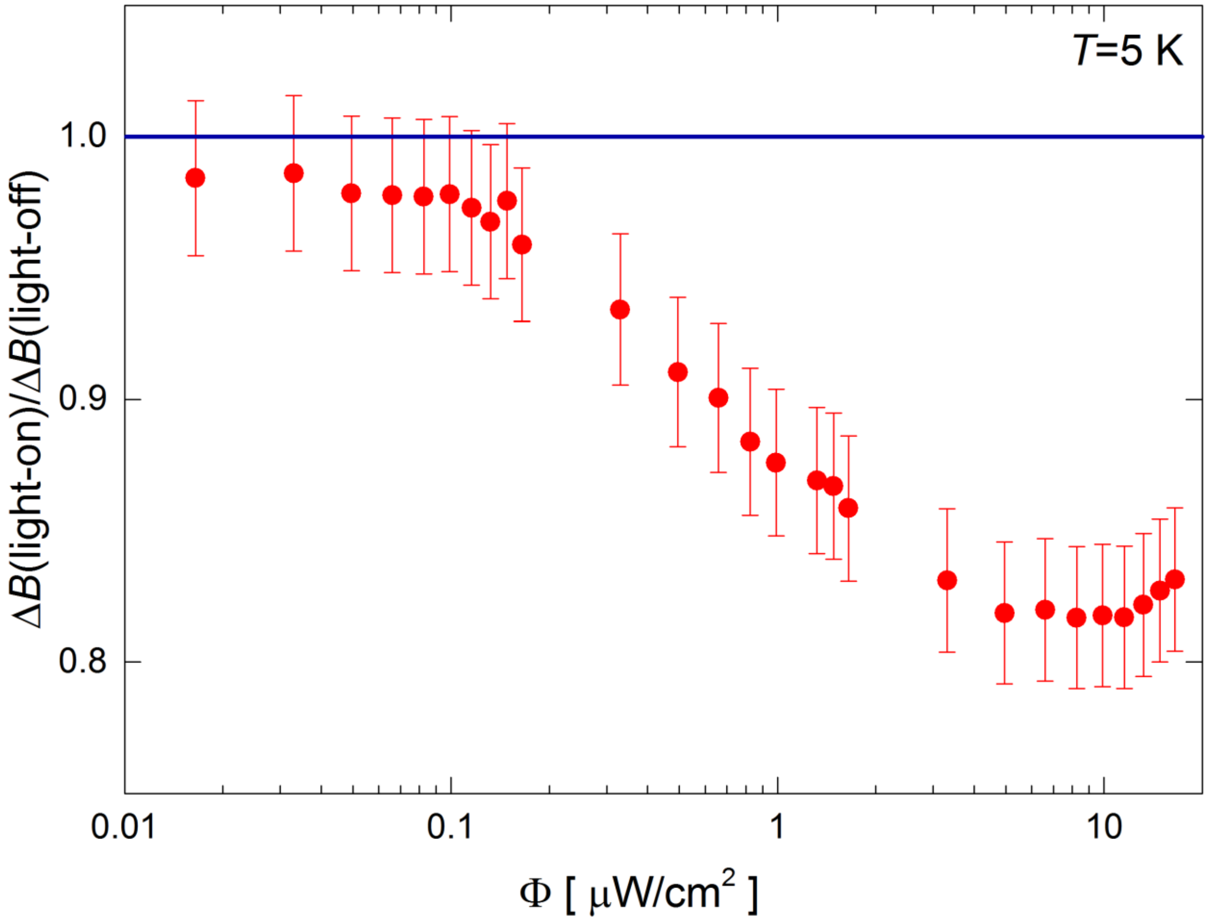

Supplementary Figure 9 - Illumination intensity effect on MAMn:PbI_3 measured by ESR. The change of the light-on ESR linewidth normalized to the linewidth in dark as the function of the illuminating red light intensity Φ at T=5 K. Above a threshold value, the FM part of the signal decreases monotonously in agreement with the intensity change seen in Fig. 3a in the main text. The error bars represent the confidence interval of least square fits to the spectra.

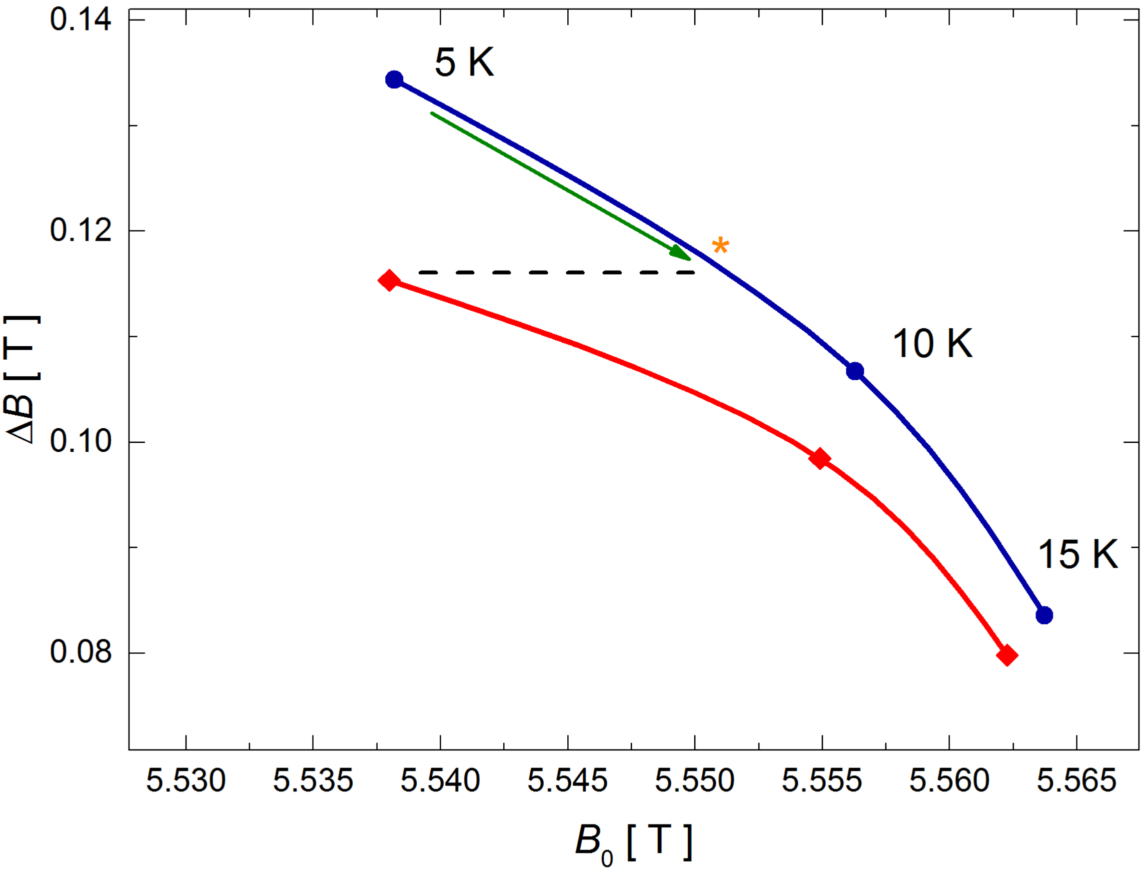

Supplementary Figure 10- Magnetization melting of MAMn:PbI$_3$: The resonance field B_0 increases with temperature, while the linewidth, ΔB of the MAMn:PbI$_3$ sample monotonically decreases (blue points are measured in dark, red points measured under 20 μW/cm^2 of light intensity at T=5, 10 and 15 K, the dashed line is a guide to the eye.) If the narrowing of ΔB was due to sample heating, one would move on the blue line in the direction indicated by the green arrow to the point shown by the orange star, and B_0 would move to a higher value.

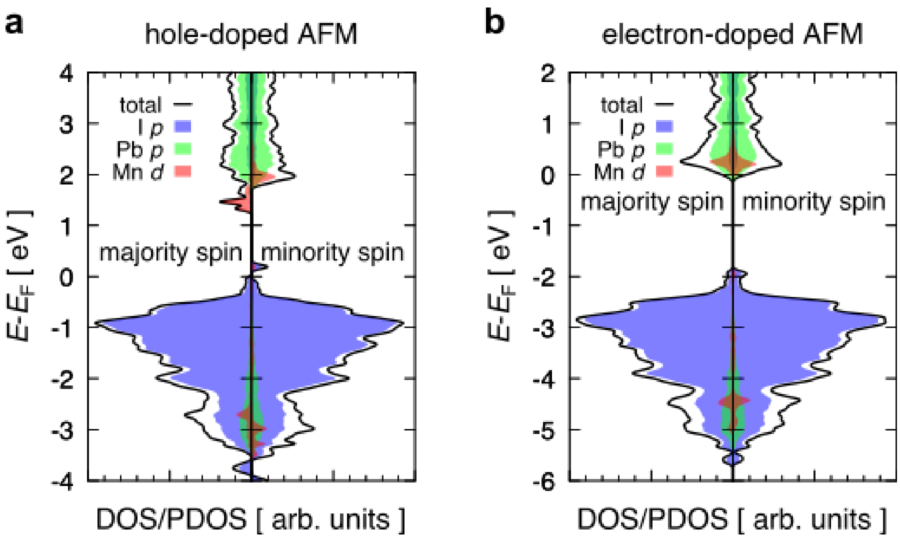

Supplementary Figure 11 – Density of states plots for doped models of MAMn:PbI$_3$. Total density of states (DOS) and projected density of states (PDOS) plots calculated from first-principles for the hole- (**a**) and electron-doped (**b**) in-plane model of MAMn:PbI$_3$ in the AFM ground state.

35

Formula	$CH_3NH_3(Pb_{0.9}Mn_{0.1})$
Cell settings	Tetragonal
Space group	*I*4*/mcm*
a (Å)	8.88078(18)
c (Å)	12.6981(3)
β (degrees)	90
**Refinement**	
R, wR (observed) (%)	2.43, 3.43
R, wR (all) (%)	2.53, 3.47
Rρ, wRρ (%)	3.11, 4.01
$\Delta\rho_{max}$, $\Delta\rho_{min}$ (e $Å^{-3}$)	0.86, -0.68

Supplementary Table 1 - Structural characteristics of MAMn:PbI_3 at 293 K
